# „Not just frailty“ – Sjögren-Syndrom und Polymyositis mit mitochondrialer Pathologie

**DOI:** 10.1007/s00393-025-01702-3

**Published:** 2025-08-22

**Authors:** Phillip Kremer, Simon Melderis, Jakob Matschke, Werner Stenzel, Ina Kötter, Martin Krusche, Marie-Therese Holzer

**Affiliations:** 1 III. Medizinische Klinik, Sektion für Rheumatologie und entzündliche Systemerkrankungen, Universitätsklinikum Hamburg-Eppendorf, Martinistr. 52, 20246 Hamburg, Deutschland; 2 Institut für Neuropathologie, Universitätsklinikum Hamburg-Eppendorf, Hamburg, Deutschland; 3 Institut für Neuropathologie, Charité-Universitätsmedizin Berlin, Corporate Member der Freien Universität Berlin, Humboldt-Universität zu Berlin, und Berlin Institute of Health (BIH), Berlin, Deutschland; 4 Klinik für Rheumatologie und Immunologie, Auenlandklinik Bad Bramstedt, Bad Bramstedt, Deutschland

**Keywords:** Kollagenose, Myositis, Frailty, Overlap-Syndrom, Mitochondriale Pathologie, Connective tissue disease, Myositis, Frailty, Overlap syndrome, Mitochondrial pathology

## Abstract

**Hintergrund:**

Das Sjögren-Syndrom (SjS) ist eine Autoimmunerkrankung aus der Gruppe der Kollagenosen, welche neben der klassischen Sicca-Symptomatik auch extraglanduläre Manifestationen aufweisen kann. Eine muskuläre Mitbeteiligung ist selten. Es existieren einige Fallberichte und Studien zur mit dem SjS assoziierten Einschlusskörpermyositis (IBM). Eine weitere assoziierte Myositisform mit mitochondrialen Veränderungen, die oft auch als Spektrum der IBM angesehen wird, ist die Polymyositis mit mitochondrialer Pathologie (PM-Mito).

**Fallbericht:**

Wir berichten den Fall einer 90-jährigen Patientin, welche sich mit progredienter Dysphagie, Gewichtsverlust und letztlich einer voranschreitenden Einschränkung beim Gehen vorstellte. In der diagnostischen Aufarbeitung konnte ein primäres SjS diagnostiziert werden, und eine Muskelbiopsie ermöglichte die Diagnosestellung einer PM-Mito.

**Schlussfolgerung:**

Der vorliegende Fallbericht verdeutlicht die Wichtigkeit, differenzialdiagnostisch an eine neuromuskuläre Mitbeteiligung im Rahmen des SjSs zu denken. Das komorbide Auftreten einer Myositis mit mitochondrialer Pathologie (PM-Mito oder IBM) mit dem SjS führt zu aktuellen Diskussionen über ähnliche pathomechanistische Aspekte. Weiterhin unterstreicht dieser Fallbericht die Wichtigkeit einer histopathologischen Beurteilung bei einer zunächst unklaren Myopathie als Schlüsselelement zur korrekten Diagnose.

## Abstract (en)

**Background:**

Sjögren’s syndrome (SjS) is a systemic autoimmune disease classified among the group of connective tissue diseases that is characterized not only by classical sicca symptoms but also by extraglandular manifestations. Muscular involvement is rare; however, several case reports and studies have described an association between SjS and inclusion body myositis (IBM). Polymyositis with mitochondrial pathology (PM-Mito) is another myositis subtype linked to SjS, featuring mitochondrial changes and often considered part of the IBM spectrum.

**Case report:**

We report the case of a 90-year-old female patient who presented with progressive dysphagia, weight loss and ultimately a progressive limitation in walking. During the diagnostic work-up, a primary SjS was diagnosed and a muscle biopsy enabled the diagnosis of PM-Mito.

**Conclusion:**

This case report highlights the importance of considering neuromuscular involvement as a differential diagnosis in patients with SjS. The comorbid occurrence of myositis with mitochondrial pathology (PM-Mito or IBM) and SjS supports ongoing discussions about similar pathomechanistic aspects. Additionally, the report underlines the crucial role of a histopathological evaluation in cases of initially unclear myopathy as the key element for a correct diagnosis.

Das Sjögren-Syndrom (SjS) ist eine autoimmunologische Systemerkrankung aus der Gruppe der Kollagenosen, welche sich neben einer klassischen Sicca-Symptomatik durch eine Vielzahl extraglandulärer Manifestation auszeichnet. In seltenen Fällen tritt das primäre SjS im Rahmen eines Overlap-Syndroms mit idiopathisch inflammatorischen Myopathien (IIM) auf, was sowohl diagnostisch als auch therapeutisch eine besondere Herausforderung darstellt. Besonders im geriatrischen Kollektiv mit zunächst unspezifischen Symptomen wie Schwäche, Dysphagie oder Gewichtsverlust kann eine entzündliche Systemerkrankung leicht übersehen werden und fälschlich als Ausdruck altersassoziierter Gebrechlichkeit („Frailty“) fehlinterpretiert werden. Zunehmende „Frailty“ im Alter betrifft über 25 % der über 75-Jährigen [1]. Frailty wird hierbei häufig definiert als Syndrom aus progredientem körperlichem Abbau, geringerer körperlicher Betätigung, Schwäche und Gewichtsverlust [2].

## Fallbericht

Eine 90-jährige Patientin, welche angab, noch vor einiger Zeit überwiegend gesund und mehrfach pro Woche sportlich aktiv gewesen zu sein, stellte sich initial mit Dysphagie, Minderung des Allgemeinzustandes sowie Gewichtsverlust in einer externen Klinik vor. Nebenbefundlich bestand seit einem Jahr eine Gangstörung bei klinisch diagnostizierter idiopathischer Polyneuropathie, welche sich zuletzt aufgrund von allgemeiner Schwäche deutlich verschlechtert habe.

Bei führender Dysphagie und Gewichtsverlust wurde eine Gastroskopie durchgeführt, in welcher sich kein wegweisender Befund ergab. In der weiterführenden Umfelddiagnostik konnte eine polyklonale Gammopathie (Immunglobulin G 56 g/l, Referenzbereich 6,5–16,0 g/l) festgestellt werden. Nachfolgend wurde eine Diagnostik mittels ANA, ENA und infektiologischem Screening initiiert. Immunserologisch imponierte ein ANA-Titer von 1:5120 (Referenzbereich < 1:80) AC‑4, mit Nachweis von stark positiven (+++, Referenzbereich *negativ*) Ro-52/60-Antikörpern sowie eine Erhöhung des Rheumafaktors auf 38 U/l (Referenzbereich < 14,0 U/l), weshalb die Patientin zur weiteren Abklärung in unsere rheumatologische Abteilung verlegt wurde.

In der detaillierten Systemanamnese wurde eine deutliche Sicca-Symptomatik mit Xerostomie angegeben. Die Kraftgradprüfung anhand des Manual-Muscle-Testing (MMT-8) [3] zeigte eine ausgeprägte proximale Muskelschwäche mit 113/150 Punkten, sodass sich die allgemeine Belastungsminderung der zuvor sportlichen Patientin als muskuläre Schwäche herauskristallisierte. Eine Schwäche der Fingerflexoren oder Knieextensoren lag nicht vor. In der klinischen Untersuchung zeigte sich ferner eine distal-symmetrische Polyneuropathie mit Pallhypästhesie (0/8 Malleolus medialis beidseits) und einem beidseitig erloschenen Patellarsehnenreflex. Elektrophysiologisch ließ sich eine sensomotorische, axonale Polyneuropathie bestätigen. Der Schirmer- und der Saxon-Test waren pathologisch, eine Speicheldrüsensonographie stellte ein inhomogenes Parenchym dar (OMERACT-Grad 2).

Laborchemisch war neben der Hypergammaglobulinämie eine deutliche Erhöhung der Kreatininkinase (1010 U/l, Referenzbereich 145 < U/l) zu verzeichnen. Der Myositisblot (16-valenter EUROIMMUN-Blot) sowie die cN-1A-Antikörper waren negativ.

**Abb. 1:**
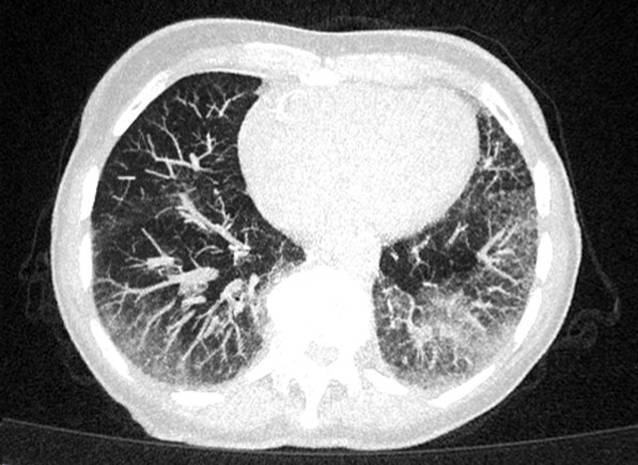
Low-dose-Computertomographie des Thorax. Bilaterale Milchglastrübungen und basale, subpleural betonte Retikulationen als Ausdruck einer interstitiellen Lungenerkrankung, radiologisch als „indeterminate UIP“ („usual interstitial pneumonia“) gewertet

**Abb. 2:**
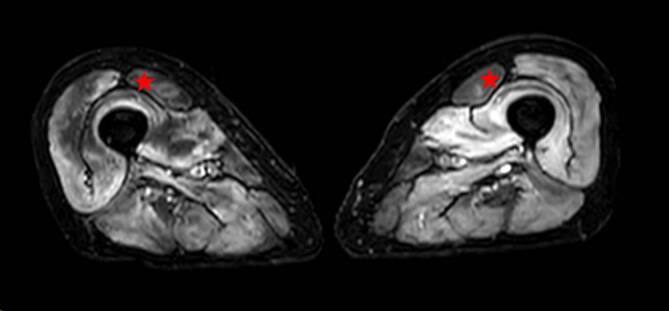
Magnetresonanztomographie der Oberschenkelmuskulatur in T2-Wichtung mit bilateralem Muskelödem, vereinbar mit Myositis. Man beachte die im Vergleich verringerte Signalanhebung des M. rectus femoris (*roter Stern*) beidseits als eines der MR-graphischen Muster der IBM [33]

**Abb. 3:**
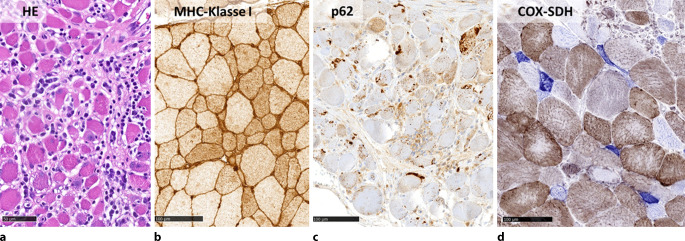
Histologie der Muskelbiopsie des M. vastus lateralis links. Histopathologisch finden sich ausgeprägte Infiltrate endomysial (**a**), welche sich immunhistochemisch vorrangig als T‑Zell-Infiltrate abbilden lassen (nicht abgebildet). Vakuolen finden sich nicht. Ferner stellt sich eine diffuse MHC-Klasse-I-Überexpression sarkolemmal und sarkoplasmatisch dar (**b**). Die p62-positiven Aggregate sind typischerweise bei IBM beschrieben ([19], **c**). Die mitochondriale Pathologie verdeutlicht sich insbesondere in der COX-SDH-Färbung, wo sich zahlreiche (über der Korrelation für den Altersdurchschnitt) COX-negative, SDH-positive Fasern darstellen (*blau* eingefärbt, **d**)

In der Computertomographie des Thorax fielen bilaterale Milchglastrübungen und subpleurale, basale Retikulationen als Ausdruck einer interstitiellen Lungenerkrankung („Indeterminate usual interstitial pneumonia“[UIP]-Muster) (vgl. Abb. 1) auf. Passend hierzu zeigte die Lungenfunktionsuntersuchung eine eingeschränkte forcierte Vitalkapazität (FVC 50 %, FEV_1_/VC 69 %). Bei hochgradigem Verdacht auf eine muskuläre Beteiligung im Rahmen einer entzündlichen Systemerkrankung erfolgte eine Magnetresonanztomographie der Oberschenkel mit Nachweis T2-hyperintenser Veränderungen der Oberschenkelmuskulatur mit geringerer Signalanhebung des M. rectus femoris beidseits (vgl. Abb. 2). Zur weiteren ätiologischen Einordnung der muskulären Beteiligung erfolgte eine Biopsie des M. vastus lateralis links. Histopathologisch waren myopathische Aspekte mit deutlicher Kalibervarianz der Muskelfasern neben diffus-endomysialen Infiltraten von autoinvasiven T‑Zellen darstellbar. Neben einer Überexpression von MHC-Klasse I und II waren zahlreiche Cytochrom-C-Oxidase(COX)-negative Muskelfasern ohne geräderte Vakuolen darstellbar, entsprechend einer Polymyositis mit mitochondrialer Pathologie (PM-Mito) (vgl. Abb. 3).

**Abb. 4:**
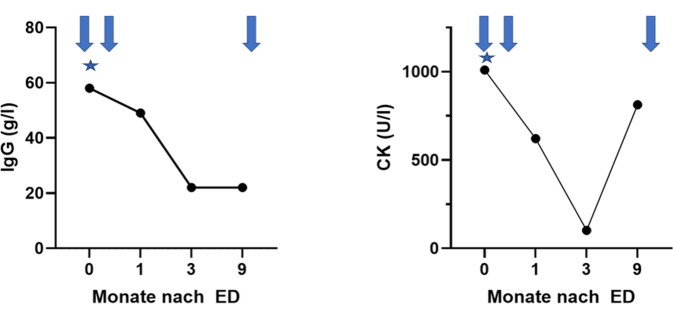
Aktivitätsparameter im Verlauf. Unter Therapie deutlicher Abfall von sowohl IgG (Monat 0: 56 g/l, Monat 1: 48,6 g/l, Monat 3: 22,8 g/L, Monat 9: 22,6 g/l) als auch CK. Parallel hierzu Anstieg des MMT‑8 von 113 (Monat 0) auf 134 (Monat 9) und eine Regredienz der Dysphagie. Neun Monate nach der letzten Rituximab-Gabe kam es unter niedrig dosiertem Prednisolon zu einem erneuten Anstieg der CK, weshalb aktuell eine Erhaltungstherapie mit Rituximab erfolgt. Die *Pfeile* markieren die Rituximab-Gaben. Der *Stern* die Immunglobulingabe (1-malig 100 g)

In der Zusammenschau stellten wir unter Hinzunahme der 2016 ACR/EULAR-Klassifikationskriterien (positive Einschlusskriterien, keine Ausschlusskriterien, Gesamt-Score 5 Punkte) die Diagnose eines klinisch hochaktiven primären SjSs (pSjS) mit pulmonaler sowie neuromuskulärer Beteiligung mit Overlap zur PM-Mito (EULAR Sjögren’s syndrome disease activity index [ESSDAI]: 23 Punkte) [4]. Aufgrund der ausgeprägten Krankheitsaktivität erfolgte eine kombinierte immunsuppressive Therapie mit Glukokortikoiden (mit nachfolgendem Ausschleichen), intravenösen Immunglobulinen (1-malig 100 g bei ausgeprägter Myopathie und Dysphagie – basierend auf den Daten der ProDERM-Studie [5]) und einer B‑Zell-gerichteten Therapie mit Rituximab (bei hoher serologischer Aktivität und schwerem klinischem Verlauf, 1000 mg an Tag 1 und Tag 15)^1^. Hierunter kam es zu einem raschen klinischen sowie laborchemischen Ansprechen (ESSDAI 4,33 im letzten Follow-up nach 9 Monaten) (vgl. Abb. 4). Der pulmonale Befund zeigte sich klinisch stabil, eine apparative Verlaufskontrolle ist als Jahres-Follow-up geplant. Zum Remissionserhalt wurde die Rituximab-Therapie aufgrund des zuvor sehr positiven Effekts bei erneutem Anstieg der CK unter niedrig dosiertem Prednisolon im Monat 10 fortgeführt (1000 mg)^1^.

## Diskussion

Der hier geschilderte Fall veranschaulicht das seltene Vorkommen einer muskulären Beteiligung im Rahmen eines SjSs mit Multiorganbeteiligung.

Durch eine umfassende Systemanamnese mit anschließender gezielter laborchemischer und apparativer Diagnostik konnte bei zunächst extern berichteter Zunahme der Frailty letztlich die Diagnose eines pSjS mit PM-Mito gestellt werden.

Das SjS ist eine inflammatorische Systemerkrankung autoimmunologischer Genese, deren häufigste Organmanifestation die Exokrinopathie mit Sicca-Symptomatik darstellt [6]. Das SjS kann isoliert oder in Assoziation mit einer Vielzahl Autoimmunerkrankungen (primär biliäre Cholangitis, Hashimoto-Thyreoiditis, entsprechend primäres SjS) oder zusammen mit anderen autoimmunologischen Systemerkrankungen (rheumatoide Arthritis, systemischer Lupus erythematodes, systemische Sklerose, dann sekundäres SjS) auftreten [7]. Über 80 % der Patient:innen leiden an einer ausgeprägten Fatigue-Symptomatik, welche die Lebensqualität nachhaltig einschränken kann [8]. Eine muskuläre Beteiligung ist beim pSjS in der Literatur nur in 1,3–3 % der Fälle beschrieben, wobei verschiedene Formen der muskulären Beteiligung vorkommen, darunter die IBM, die PM-Mito und die Overlap/Poly-Myositis [9]. In der Kohortenstudie von Colanfancesco et al. [9] zeigten die untersuchten Muskelbiopsien insbesondere Aspekte einer „Polymyositis“ oder IBM, wobei knapp die Hälfte der Muskelbiopsien mitochondriale Veränderungen im Sinne von vermehrten COX-negativen Fasern aufwiesen. Eine 2021 publizierte Registerstudie berichtete bei 50 % der Myositis-SjS-Overlap-Syndrome die Diagnose einer IBM [10]. Ferner lässt sich bei rund 6–10 % der Patient:innen mit einer IBM ein komorbides SjS finden [11, 12]. Die dezidierte Unterscheidung der muskulären Beteiligung bringt unmittelbare therapeutische Implikationen mit sich, da PM-Mito-Patient:innen in Fallberichten besser auf eine immunsuppressive Therapie (wie beispielsweise mit Glukokortikoiden, Methotrexat, Mycophenolat-Mofetil) anzusprechen scheinen als IBM-Patient:innen [13]. Sowohl die muskuläre Beteiligung als auch die Polyneuropathie und Fatigue können bei unserer Patientin einen wesentlichen Anteil der initialen Symptomatik verursacht haben. Die Dysphagie, die sich unter Therapie deutlich besserte, kann als Folge der deutlichen Xerostomie, aber auch als Manifestation im Rahmen der Myositis gewertet werden. Sowohl PM-Mito- als auch IBM-Patient:innen können eine Dysphagie entwickeln.

**Abb. 5:**
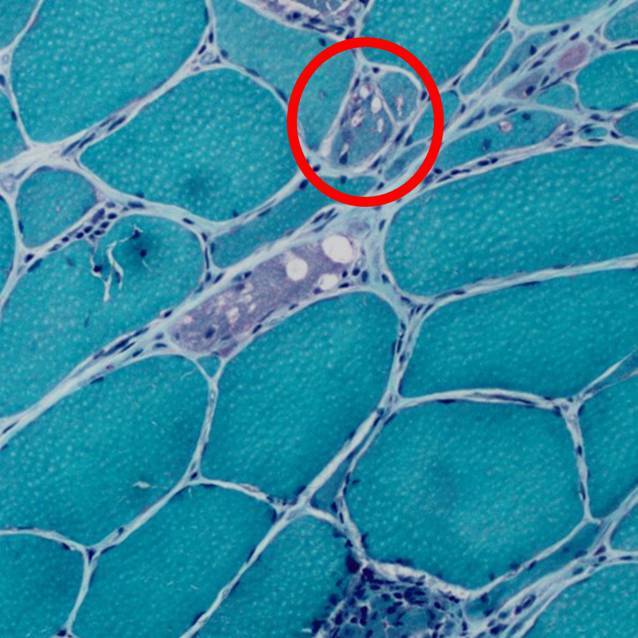
Vergleichsabbildung einer Muskelbiopsie eines IBM-Patienten (Gömöri-Färbung). Eingekreist sind die klassischen „rimmed vacuoles“, die einen wichtigen Unterscheidungspunkt von IBM-Patient:innen und PM-Mito-Patient:innen darstellen („rimmed vacuoles“ können prinzipiell auch bei anderen Muskelerkrankungen auftreten und sind alleine betrachtet nicht pathognomonisch für die IBM)

Die sporadische Form der IBM stellt die häufigste Myopathie im fortgeschrittenen Alter (Krankheitsbeginn typischerweise > 40 Jahre) dar. Sie ist durch eine Beteiligung der distalen Muskulatur (insbesondere Fingerbeuger- und Kniestreckermuskulatur), einen langsamen Krankheitsprogress sowie ein geringes therapeutisches Ansprechen gekennzeichnet [14–16]. Die PM-Mito wurde erstmals 1997 beschrieben und stellt eine Verlaufsform der idiopathisch inflammatorischen Myopathien dar [17]. In den vergangenen Jahren wurde diese genauer charakterisiert [18, 19]. Die PM-Mito und die IBM weisen eine Vielzahl klinischer, histologischer und molekularer Gemeinsamkeiten auf, sodass die PM-Mito zunehmend als frühe Verlaufsform der IBM im Sinne einer Spektrum-Erkrankung eingeordnet wird („early IBM“ [eIBM]). Histologisch kennzeichnet sich die IBM durch endomysiale Inflammation mit Invasion von CD8^+^-T-Zellen und MHC-Klasse-I-Expression auf nichtnekrotischen Muskelfasern sowie Merkmale einer mitochondrialen Schädigung, darunter Cytochrom-C-Oxidase(COX)-defiziente Fasern, „ragged red fibers“ (RRFs) und eine abnorme Proteinakkumulation [16, 17, 20, 21]. Histopathologisch kann eine weitere Abgrenzung der IBM gegenüber anderen Entitäten mittels immunhistochemischer Färbung gegen TDP43 und p62 erfolgen [22]. In Abgrenzung zur sporadischen IBM fehlen bei der PM-Mito die charakteristischen „rimmed vacuoles“ (Abb. 5) sowie in der Regel auch Proteinaggregate mit immunhistochemischer Reaktivität für TDP43 oder p62 [17–19]. Auf pathophysiologischer Ebene scheint eine abnorme Interferon-mediierte Inflammation sowohl bei der IBM als auch der PM-Mito eine Rolle zu spielen. Bei Patient:innen, die initial mit einer PM-Mito diagnostiziert wurden, entwickelte nach Kleefeld et al. im weiteren Krankheitsverlauf ein Großteil (13 von 14 Patient:innen, 93 %) eine definitive IBM [19].

In der Pathogenese des pSjS und der IBM sind ebenfalls einige Parallelen zu erkennen [23]: So spielt eine aberrante T‑Zell-Antwort mit Nachweis autoreaktiver CD4^+^- oder CD8^+^-T-Zellen eine Schlüsselrolle [24, 25]. Weiterhin legen Daten eine gemeinsame genetische Prädisposition in Assoziation mit dem humanen Leukozytenantigen(HLA)-Allel *HLA-DR3 *nahe [26]. Der Nachweis von Anti-cN-1A-Antikörpern, welcher ein unterstützendes diagnostisches Kriterium für die IBM darstellt [16], lässt sich zudem bei ca. 12 % der Patient:innen mit SjS erbringen [27]. Eine kürzlich veröffentlichte Arbeit von Levy et al. legt jedoch nahe, dass bei Myositis-Patient:innen der Zusammenhang zwischen Anti-cN-1A und SjS unabhängig vom Zusammenhang zwischen IBM und SjS ist [28, 29].

Fallberichte zu pSjS und PM-Mito liegen nur sehr wenige vor. So berichteten z. B. Papadimas et al. von einer 38-jährigen Patientin, die im Verlauf gut auf eine Immunsuppression mit Prednisolon und Methotrexat ansprach [13].

Auch eine neurologische Beteiligung beim pSjS kann sich in muskulärer Schwäche oder Gangstörungen äußern. Der peripheren neurologischen Beteiligung können verschiedene Ursachen, wie z. B. eine Small-Fiber-Neuropathie oder eine vaskulitische Beteiligung beim pSjS zugrunde liegen [30]. Aufgrund der eindeutigen klinischen Situation und fehlender therapeutischer Konsequenz wurde bei unserer Patientin keine Nerven- oder Hautbiopsie durchgeführt. Die ILD unserer Patientin ist ebenfalls dem pSjS zuzuordnen, bei dem eine Lungenbeteiligung in 10–20 % der Fälle auftreten kann. Am häufigsten sind hierbei die „non-specific interstitial pneumonia“ (NSIP), gefolgt von der UIP und lymphozytären interstitiellen Pneumonie (LIP) zu verzeichnen [31]. Gerade bei schweren oder therapierefraktären Fällen der ILD beim pSjS oder anderen schweren Organmanifestationen wird Rituximab als Therapieoption diskutiert [31, 32].

Die Diagnose der PM-Mito bei unserer Patientin fußte auf der Klinik bestehend aus proximaler Muskelschwäche ohne Zeichen für eine Fingerflexor- oder Knieextensorschwäche sowie dem typischen histopathologischen Erscheinungsbild ohne „rimmed vacuoles“, wobei beginnende p62-Aggregate bereits als Zeichen einer „early IBM“ diskutiert werden können ([19], vgl. Abb. 4). Ferner ist die relative Aussparung des M. rectus femoris ein ebenfalls bei der IBM beobachtetes Phänomen [33]. Das erfreuliche Ansprechen auf Immunsuppression unterstützt die Diagnose der PM-Mito bei unserer Patientin.

Zusammenfassend berichten wir von dem sehr seltenen Fall eines primären SjSs mit zeitgleich vorliegender PM-Mito. Insbesondere im fortgeschrittenen Alter können unspezifische, mit dem Frailty-Syndrom überlappende Symptome die Diagnosestellung erschweren. Eine muskuläre Beteiligung beim SjS sollte daher bedacht und differenzialdiagnostisch eruiert werden. Eine histopathologische Untersuchung kann insbesondere hier Aufschluss geben über die Genese der Myositis beim pSjS, die häufig von mitochondrialer Pathologie geprägt ist.

## Fazit für die Praxis

- Das primäre Sjögren-Syndrom (SjS) kann sich initial unspezifisch äußern und gerade im Alter eine diagnostische Herausforderung darstellen.
- Die Diagnose einer Polymyositis mit mitochondrialer Pathologie (PM-Mito) bringt therapeutische und prognostische Implikationen mit sich und sollte differenzialdiagnostisch auch bei initial unspezifischer Symptomatik in Betracht gezogen werden.
- Die PM-Mito ist bei Patient:innen mit SjS eine sehr seltene Manifestation, häufiger wird die Diagnose einer IBM gestellt.
- Die Dysphagie bei SjS kann sowohl Ausdruck der Xerostomie als auch einer muskulären Beteiligung sein.

## Footnotes

1 Anmerkung: Der Einsatz von Rituximab erfolgt beim pSjS als Off-label-Therapie. Die Applikation bei unserer Patientin wird aufgrund des fortgeschrittenen Alters und der bisher fehlenden Studien im Bereich PM-Mito-Sjögren-Syndrom-Overlap individuell anhand der Klinik gesteuert.
